# The imaginary divide between mental and “physical” health: Dismantling dualism and reductionism to address a monumental mistake in medicine

**DOI:** 10.1002/jcv2.70123

**Published:** 2026-05-11

**Authors:** Nicholas Fabiano, Marco Solmi, Kurt Kroenke, Brendon Stubbs, Joseph Firth, Fiona Gaughran, Katarzyna K. Machaczek, Christopher Palmer, Jess G. Fiedorowicz

**Affiliations:** ^1^ Department of Psychiatry University of Ottawa Ottawa Ontario Canada; ^2^ Department of Mental Health Ottawa Hospital Ottawa Ontario Canada; ^3^ Ottawa Hospital Research Institute (OHRI) University of Ottawa Ottawa Ontario Canada; ^4^ School of Epidemiology and Public Health Faculty of Medicine University of Ottawa Ottawa Ontario Canada; ^5^ Department of Child and Adolescent Psychiatry Charité Universitätsmedizin Berlin Germany; ^6^ Department of Medicine Regenstrief Institute Indiana University School of Medicine Indianapolis Indiana USA; ^7^ Clinical Division of Social Psychiatry, Department of Psychiatry and Psychotherapy Medical University of Vienna Vienna Austria; ^8^ Comprehensive Centre for Clinical Neurosciences and Mental Health (C3NMH) Medical University of Vienna Vienna Austria; ^9^ Psychological Medicine Institute of Psychiatry, Psychology and Neuroscience (IoPPN) King's College London London UK; ^10^ Division of Psychology and Mental Health Manchester Academic Health Science Centre, University of Manchester Manchester UK; ^11^ Greater Manchester Mental Health NHS Foundation Trust Manchester Academic Health Science Centre Manchester UK; ^12^ Department of Psychosis Studies Institute of Psychiatry, Psychology and Neuroscience Kings College London London UK; ^13^ National Psychosis Service South London and Maudsley NHS Foundation Trust London UK; ^14^ Advanced Wellbeing Research Centre and Centre for Applied Health and Social Care Research Sheffield Hallam University Sheffield UK; ^15^ Metabolic and Mental Health Program McLean Hospital Belmont Massachusetts USA; ^16^ Psychiatry Harvard Medical School Boston Massachusetts USA

**Keywords:** access to health care, biopsychosocial, coordinated services, dualism, mental, mental health, physical, psychiatric disorder, reductionism

## Abstract

Given the importance of the link between mental and other medical conditions, *JCPP Advances* organized a special issue on the topic; yet since then, very few papers have focused on this area. As such, this editorial perspective aims not only to highlight the link between mental and other medical conditions, but also to (1) explore the origins of the divide between mental and “physical” health, (2) provide evidence that this so‐called divide does not exist in actuality, (3) highlight the harms of maintaining such a divide, and (4) discuss strategies to bridge this divide to address this monumental mistake, which has been perpetuated throughout medicine.

## Key Points

**What's known?**

- Modern medicine has adopted the superficial idea that mind and body are separate, and this view has become entrenched, costly and an impediment to progress.

**What's new?**

- We explore the origins of the divide between mental and physical health, provide evidence that this so‐called divide does not reflect reality, highlight the harms of maintaining such a divide, and discuss strategies to bridge this divide.

**What's relevant?**

- For medicine to progress, we must expunge ourselves of dualist language and frameworks, broaden our approach, and redesign our systems of care.

## THE ORIGIN AND PERPETUATION OF THE DIVIDE

The divide between so‐called mental and physical health was crystalized in the seventeenth century by the French philosopher René Descartes. He proposed the concept of “mind‐body dualism,” in which the mind (or soul) was regarded as a non‐physical thinking substance that was entirely separate from the body, which in turn was a mechanical, physical entity governed by natural laws. Therefore, Descartes distinguished mind and body ontologically as two separate spheres of being. Yet, he simultaneously emphasized that they constituted a unity in the case of the human being, and he claimed that many diseases had psychosomatic causes and cures.

Unfortunately, modern medicine has adopted the superficial idea that mind and body are separate (an imprecise extension of Descartes' work) and this view has become entrenched, costly and an impediment to progress. Notably, modern medicine tends to follow a reductionist approach, with a focus on biological signs, symptoms, and treatments, with a minimal focus on or even marginalization of psychosocial causal or perpetuating factors. Such a narrow focus can be myopic and miss the broader picture in a potentially dehumanizing manner. This approach prioritizes the biologically explainable aspects of disease presentations and impedes recognition and treatment of components not easily reduced to biological mechanisms, which has created stigma around mental health and associated disparities in care. Beyond this, medical education and training generally operate in silos so that physicians with clinical care and training seldom integrate psychiatry with other areas of medicine (Fabiano et al., 2022).

Attempts have been made to bridge this gap and avoid such reductionism. For instance, George Engel's biopsychosocial model advocates the simultaneous consideration of biological, psychological, and social factors as root causes of health conditions, including mental (Engel, 1977). Despite this, dualism persists, and reductionism remains rampant. To address this divide, we must change our language, redesign care pathways, and realign incentives to support integrated, multidisciplinary practice.

## THE DIVIDE IS UBIQUITOUS ALTHOUGH NOT USEFUL

This arbitrary schism between mental and “physical” health has propagated into other dubious dualities, such as psychosomatic, physical‐psychological, mental‐physical, and biomedical‐psychosocial, and objective‐subjective, inter alia. Theoretically, we can separate perspectives and means of understanding while still integrating phenomena whether medically explained or unexplained or in the traditional domain of mental health or other medical domains. Mental health is part of health, just as cardiovascular health and skin health are. However, unlike cardiovascular health, mental health has been plagued by its juxtaposition with “physical” health. To address this issue, we must revisit the professional usage of the term “physical health” as a juxtaposition to mental health, and with it the harmful assumptions of dualism and the biological reductionism it instils.

In many cases, mental and other medical conditions are systemic in nature and not isolated to one organ. Comorbidity, including between mental and other medical conditions, is the rule rather than the exception. Population‐based studies have demonstrated bidirectional relationships between mental disorders and other medical conditions (Momen et al., 2020, 2024). There now exists evidence of a common factor that underlies mental and other health conditions—the general disease (“d”) factor (Brandt et al., 2023; Cortese et al., 2021). All health conditions share common biopsychosocial risk factors, including genetic vulnerabilities, health‐risk behaviors (such as poor diet, smoking, and low physical activity) and associated socioeconomic factors. They also share important pathophysiological pathways, including inflammation, mitochondrial and metabolic dysfunction, hypothalamic‐pituitary‐adrenal axis dysregulation, and changes in the gut microbiome, although these can manifest differently among individuals, as well as across diagnoses. Recognizing these shared causalities presents opportunities to improve prevention and treatment strategies.

One mental disorder in particular highlights the need for an integrated approach. Over 290 million people worldwide have been diagnosed with major depression, making it one of the most prevalent mental disorders. In a study of 1146 people with major depression across 15 countries, 45%–95% reported bodily symptoms only (e.g., headache, constipation, weakness, or back pain), while 11% denied having any of the psychological symptoms of depression, even when directly questioned, until they underwent structured diagnostic interviews (Simon et al., 1999). Mental disorders are not limited to psychological symptoms and vice versa (Bonthrone et al., 2024). Indeed, somatic symptoms that are typically attributed to cardiac, pulmonary, endocrine and rheumatological diseases can be influenced as much by depression and anxiety as by the severity of the underlying medical disorder itself. Treatments for depression also vary from those that target processes at a biological level of understanding (e.g., medications) to those that act at a psychological level (e.g., psychotherapy). Treatments that promote general health, such as exercise, are equally effective (Fabiano et al., 2025).

There are many common examples of medical conditions that highlight the need to integrate the approaches. Back pain affects over 619 million people, and is the leading global cause of years lived with disability. From a biological perspective, one would imagine that high‐resolution magnetic resonance imaging (MRI) of the spine could be used as an accurate predictor of the incidence of back pain. However, a prospective cohort study of 148 Veterans Affairs outpatients found that MRI did not predict the 3‐year incidence of back pain, although diagnosis of depression did (hazard ratio [2.3, 95% CI = 1.2–4.4]) (Jarvik et al., 2005). Further, only 1 in 10 biological treatments for back pain are efficacious, while psychological treatment (via pain reprocessing therapy) has demonstrated significant pain improvement, with functional MRI correlates of reduced responses to evoked back pain in the anterior midcingulate and the anterior prefrontal cortex (Ashar et al., 2022). Thus, dualistic assumptions and biologically or psychologically reductionistic positions do not reflect the realities of medicine. An integrated approach with multiple perspectives to understanding offers a broader, more integrated approach to clinical management and future research.

## THE HARMS OF THE ARTIFICIAL DIVIDE

This artificial divide between mental and physical health harms patients. First, the divide has resulted in a two‐tier system, in which mental health is perceived as “less real.” This stigma manifests in healthcare, in which people with mental disorders receive lower‐quality treatment than do those with other ailments, or treatment via diagnostic overshadowing, in which somatic symptoms are attributed solely to a mental illness origin. On a larger scale, this discrimination has resulted in disproportionate underfunding of mental health services and research. As a result, fewer resources are made available, so wait times increase, and treatment pathways become unequal. The downstream effects of underinvestment in mental health provision are readily quantifiable in mortality rates, as inadequately or untreated mental illness impacts the ability to access unmodified healthcare pathways (Chan et al., 2023; Solmi et al., 2020). For example, a meta‐analysis of 4,717,839 people found that, despite increased mortality rates from cancer among people with mental disorders, fewer of this group of people were screened (Odds ratio 0.76 [95% CI = 0.72–0.79]) compared with the general population (Solmi et al., 2020). Similar disparities exist in the treatment of cardiovascular conditions in persons with mental health conditions (Gupta et al., 2026). Such disparities contribute to the significant life expectancy reduction of 15 years for people with mental health conditions compared with the general population, all of which are exacerbated among marginalized groups (Chan et al., 2023).

Beyond the direct harm to patients, this siloed approach to healthcare has systemic and economic implications. Mental health inequalities in the United States (US) alone currently cost approximately $477.5 billion annually, which is expected to increase to nearly $14 trillion by 2040, equating to an estimated $42,000 per person living in the US (Dawes et al., 2024). These expenditures largely arise from the exacerbation of other chronic diseases (e.g., diabetes mellitus, cardiovascular disease, HIV), emergency department overutilization, productivity‐related losses stemming from absenteeism and unemployment, and premature death. An integrated healthcare policy, with an emphasis on prevention, early identification, and concomitant disease management, has the significant potential to lower these costs and improve healthcare outcomes for all.

## HOW TO BRIDGE THE DIVIDE

As the partitioning of mental from physical health is based on tenuous assumptions, we must retire the notion of a broad separation between “physical” and mental: health that pervades the academic spheres and clinical care systems that aim to understand and treat health conditions. “Mental health awareness” initiatives would become more informative if they acknowledged physiological elements and the overlap of mental health conditions with others. In terms of medical education—through medical school to residency and beyond, we must train clinicians to recognize the profound interconnectedness between mental and overall health, and the need to treat disease holistically. Regardless of their respective medical specialties, healthcare professionals must either be cross‐trained to provide comprehensive care or more realistically foster integration and collaboration between specialists. It is known that mortality risk is greater if a mental illness is left untreated (Chan et al., 2023). Therefore, in psychiatry, the aim should be to treat people with the attention required to ensure that they are placed in the best position possible to address their broader health needs, be they acute or long‐term, while minimizing the risk or impact of adverse effects. Psychiatry must also, while maintaining expertise in mental status of behavior, assume some responsibilities pursuant to a broader medical training and protect against defining the field as specializing in that which is biologically unexplained or idiopathic.

This integration must extend to practice models, including behavioral health in primary care, team‐based approaches that include lifestyle medicine, and coordinated attention to nutrition, sleep and exercise, with consideration of socioeconomic determinants. For this to succeed, payers and health systems alike must support comprehensive, multidisciplinary care rather than the extant, fragmented reimbursement models. Both policy and systemic changes are imperative. As of March 2025, the World Health Organization has called for urgent transformation of mental health policies to include promotion of holistic care with an emphasis on lifestyle, general health, and social, and economic interventions. It is envisaged that these should address those socioeconomic factors that shape health (e.g., employment, housing and education); and implement prevention strategies that promote population‐wide health as a whole.

High‐quality research that is focused on the overlap between mental and other medical conditions is urgently needed to move this space forward. This must focus on disparities in care, predictors of adverse health outcomes in those with mental disorders, and interventions that can close the gap between mental and other health outcomes (Solmi et al., 2020, 2024).

While removing the divide is imperative, enhancing health is not just the responsibility of health sectors but an overall systems goal. The continued use of the terms “mental” and “physical” health has perpetuated this divide; and we must instead utilize inclusive terminology that encapsulates health as a whole, such as “one health,” “whole health,” “mental and other medical conditions,” “integrated well‐being,” “holistic health,” among others. For medicine to progress, we must expunge ourselves of dualist language and frameworks, broaden our approach, and redesign our systems of care.

## AUTHOR CONTRIBUTIONS

**Nicholas Fabiano**: Conceptualization; writing—original draft; writing—review and editing. **Marco Solmi**: Writing—review and editing. **Kurt Kroenke**: Writing—review and editing. **Brendon Stubbs**: Writing—review and editing. **Joseph Firth**: Writing—review and editing. **Fiona Gaughran**: Writing—review and editing. **Katarzyna K. Machaczek**: Writing—review and editing. **Christopher Palmer**: Writing—review and editing. **Jess G. Fiedorowicz**: Writing—review and editing; conceptualization; supervision.

## CONFLICT OF INTEREST STATEMENT

M.S. reports honoraria and consulting fees from Angelini, AbbVie, Bausch Health, Boehringer Ingelheim, Lundbeck, Otsuka, and Teva. B.S. is on the Editorial Board of the *Journal of Physical Activity and Health*, *Aging Research Reviews*, *Mental Health and Physical Activity*, *The Journal of Evidence Based Medicine*, and *The Brazilian Journal of Psychiatry*. B.S. is supported by an NIHR Advanced Fellowship (NIHR301206) and has received honorarium from a co‐edited book on exercise and mental illness (Elsevier), an associated education course and unrelated advisory work from ASICS and FitXR LTD. J.F. is supported by a University of Manchester Presidential Fellowship (P123958) and a UK Research and Innovation Future Leaders Fellowship (MR/T021780/1) and has received honoraria/consultancy fees from Atheneum, Informa, Gillian Kenny Associates, Big Health, Nutritional Medicine Institute, ParachuteBH, Richmond Foundation and Nirakara, independent of this work. F.G. reports honoraria from BMS, Boehringer Ingelheim, Lundbeck, Recordati, and has a family member with previous professional links to Lilly and GSK. F.G. is in part supported by the National Institute for Health Research's (NIHR) Biomedical Research Center at South London and Maudsley NHS Foundation Trust and King's College London; and the NIHR Applied Research Collaboration South London at King's College Hospital NHS Foundation Trust; and by the Medical Research Council [MR/Z503563/1]; as part of Hub for Metabolic Psychiatry, one of the 6 Hubs comprising the UKRI Mental Health Platform [MR/Z000548/]. The views expressed are those of the author(s) and not necessarily those of the funders, including NIHR or the Department of Health and Social Care. K.K.M. is supported partially by National Institute for Health and Care Research (NIHR) grants NIHR201618 and NIHR206943. The remaining authors have declared that they have no competing or potential conflicts of interest.

## ETHICAL CONSIDERATIONS

Not applicable.

## Data Availability

Data sharing not applicable to this article as no datasets were generated or analyzed during the current study.
